# Transcriptome analysis in primary colorectal cancer tissues from patients with and without liver metastases using next‐generation sequencing

**DOI:** 10.1002/cam4.1147

**Published:** 2017-07-26

**Authors:** Sen Wang, Chuan Zhang, Zhiyuan Zhang, Wenwei Qian, Ye Sun, Bing Ji, Yue Zhang, Chunyan Zhu, Dongjian Ji, Qingyuan Wang, Yueming Sun

**Affiliations:** ^1^ The First Clinical Medical College Nanjing Medical University Nanjing Jiangsu 210029 China; ^2^ Department of Colorectal Surgery The First Affiliated Hospital of Nanjing Medical University Nanjing Jiangsu 210029 China

**Keywords:** Colorectal cancer, CRC, liver metastases, next‐generation sequencing, NGS

## Abstract

Colorectal cancer (CRC) is the third most common cancer worldwide and liver metastases are the leading cause of death in patients with CRC. In this study, we performed next‐generation sequencing profiling on primary colorectal tumor tissues obtained from three CRC patients with liver metastases and three CRC patients without liver metastases to identify differentially expressed genes (DEGs) that might be responsible for the metastases process. After filtering 2690 DEGs, comprising 996 upregulated and 1694 downregulated RNAs, 22 upregulated and 73 downregulated DEGs were identified. Gene ontology (GO) and pathway analyses were performed to determine the underlying mechanisms. Single‐organism process (biological process), cell (cellular component), and binding (molecular function) were the most related terms in the GO analysis. We selected the top 13 upregulated and top 12 downregulated genes by fold change to verify their differential expression using quantitative real‐time reverse transcription PCR (qRT‐PCR) and immunohistochemistry (IHC). The validation showed that three most significantly upregulated DEGs were *HOXD10*,*UGT2A3*, and *SLC13A2,* whereas the five most significantly downregulated DEGs were *SPP1*,*CXCL8*,*MMP3*,*OSM*, and *CXCL6*, respectively. These aberrantly expressed genes may play pivotal roles in promoting or inhibiting metastases. Further studies are required to determine the functions of DEGs to promote the diagnosis of metastases and provide novel chemotherapy targets.

## Introduction

Colorectal cancer (CRC) is the third most common type of cancer globally, resulting in 700,000 deaths annually, and this number is still increasing 1. The liver is the most frequent distant metastases organ for CRC, and liver metastases are the leading death cause for CRC patients 2. Approximately 10–15% of patients are diagnosed with CRC with synchronous liver metastases; however, only 10–20% of patients qualify for surgery, which it is the only possible treatment to cure CRC with liver metastases currently 3. Unfortunately, about 30–50% of patients would suffer from reoccurrence of local tumor or distant metastasis even after curative resection of primary tumors 4, 5. Liver metastases are a multiple‐step process influenced by various factors, and is not just the simple process whereby cells migrate from primary lesions to distant organs. Given our poor understanding of genetic factors that affect this process, it is critically important to reveal the genetic and molecular causes that underlie the occurrence of liver metastasis. Meanwhile, conventional methods to detect distant metastases are still radiology and biopsy, which lack economic efficiency and convenience 6. Thus, novel biomarkers of metastatic progression are required urgently to detect liver metastases and improve diagnostic accuracy.

The emergence of next‐generation sequencing (NGS) has already changed the ways we perform genomic studies. Before NGS, gene expression was quantified by other methods, such as hybridization‐based microarrays. Compared with microarrays, NGS is able to detect RNAs with very low expressions and identify unrecognized novel targets 7, as well as providing more detailed information 8. Several previous studies 9, 10, 11, 12, 13, 14, 15, 16 used NGS to seek mutations and differentially expressed genes (DEGs) that could play pivotal roles in the colorectal carcinogenesis and metastases, which reveals the significance of NGS.

However, although NGS has been applied in studies involving CRC liver metastases, it was reported that metastatic and primary CRC demonstrated a high concordance in gene expression. Therefore, our study adopted another strategy. Comprehensive NGS‐based whole‐transcriptome profiling was performed on six primary colorectal cancer tissues obtained from three patients with liver metastases and three patients without liver metastases. Libraries of total RNA and small RNAs were constructed for sequencing. DEGs were examined and filtered after sequencing. Our goal was to identify DEGs that might be vital in regulating the process of metastases and potential target biomarkers through next‐generation RNA‐seq technology.

## Materials and Methods

### Patients and samples

We adopted some methods to screen all the samples from the biobank in the Department of Colorectal Surgery, the First Affiliated Hospital of Nanjing Medical University, between 2014 and 2016. First, we divided these samples into two categories: one is primary CRC samples with liver metastases and the other is primary CRC samples without liver metastases. Then, to decrease potential discordance, only T4 samples (according to TNM staging) were collected from patients with only left colon tumors who were enrolled in this study. Finally, samples must be obtained from patients without receiving neoadjuvant chemotherapy. Through this filtering process, 36 fresh primary CRC samples were collected and then we performed quality tests twice on all 36 samples before next‐generation RNA‐seq. After that, three primary CRC samples with liver metastasis and three primary CRC samples without metastasis (i.e., six primary CRC samples) were subjected to NGS. We therefore performed NGS on these qualified samples. All the patients and grouping information are listed in the Table 1. All these samples were confirmed as pathologically adenocarcinoma. All samples were snap‐frozen in the liquid nitrogen after surgery 15 and stored at −80°C within 30 min after excision of the tumor. Written informed consent was obtained from all patients, and this study was approved by the Ethics Committee of the First Clinical Medicine College, Nanjing Medical University.

**Table 1:** Information for patients whose tissue were obtained for next‐generation sequencing (NGS)‐based profiling

No.	Group	Age	Gender	Location	Pathology	Metastases	Stage	Primary tumor size (cm)
T2	T	62	M	Left colon	Adenocarcinoma	Liver	T4N2M1	4*3*1.5
T8	T	57	M	Left colon	Adenocarcinoma	Liver	T4N1M1	7*6*2
T9	T	54	F	Left colon	Adenocarcinoma	Liver	T4N2M1	4.5*2.5*0.5
T4	C	57	M	Left colon	Adenocarcinoma	No	T4N0M0	6*4*2
T6	C	59	M	Left colon	Adenocarcinoma	No	T4N0M0	6*6*1.5
T7	C	62	M	Left colon	Adenocarcinoma	No	T4N0M0	3*2*1

### RNA extraction

Total RNA was isolated from each sample using the TRIzol reagent (Invitrogen, Carlsbad, CA), following the manufacturer's protocol. The purified RNA samples were quantified using a NanoDrop 2000 spectrophotometer (Agilent, Santa Clara, CA). The isolated RNA was stored at −80°C until analysis. After screening all 36 RNA samples using the Agilent 2100, six of them met the level A standard for subsequent sequencing. The RNA Integrity Number (RIN) was confirmed as above 7.0 and rRNA 28S/18S ratio was above 1.0 separately in these six samples.

### Illumina‐based sequencing

The total RNA samples were first treated with DNase I to degrade any possible DNA contamination. Oligo(dT) magnetic beads were then used to enrich the mRNA. The mRNA was fragmented into short fragments after mixing with fragmentation buffer. Using random hexamer‐primers, first strand cDNA was synthesized. The second strand was synthesized using buffer, dNTPs, RNase H, and DNA polymerase I. The double strand cDNAs were purified with magnetic beads. Finally, sequencing adaptors were ligated to the fragments. PCR amplification then enriched the fragments. The library products were ready for sequencing using the Illumina HiSeq^™^ 2000 system.

### Quality control and filtering

Primary sequencing data produced by the Illumina HiSeq^™^ 2000 were termed raw reads. “Dirty” raw reads were defined as those that contained the sequence of the adaptor, high content of unknown bases, and low‐quality reads. These dirty reads were filtered out as follows: (1) Reads with adaptors were removed; (2) reads in which unknown bases were more than 10% were removed; and (3) low‐quality reads (the percentage of low‐quality bases was over 50% in a read) were removed. All subsequent analyses were based on the clean data.

### Gene quantification

To quantify each DEG, the fragments per kb per million fragments (FPKM) method are adopted 17. To calculate the expression level, the following formula was used:

$$\text{FPKM}=\frac{10^{6}C}{\text{NL}/10^{3}}$$

In this formula, Given the gene A, C represents the number of fragments that are uniquely mapped to gene A, *N* is the total number of fragments that are uniquely mapped to all genes, and L is the number of bases of gene A. The FPKM method can eliminate the influence of different gene length and sequencing discrepancy on the calculation of gene expression. Therefore, the calculated gene expression can be used directly for comparisons between samples.

### Data analysis

*P*‐value and false discovery rate (FDR) filtering was used to identify the mRNAs and small RNAs whose expression was significantly different between Treatment (T) Groups (T2, T8, and T9) and Control (C) Groups (T4, T6, and T7). These differentially expressed mRNAs and small RNAs were identified through fold change filtering.

### Gene function and Kyoto Encyclopedia of Genes and Genomes pathway enrichment analysis

Gene Ontology (GO) is an international standard gene functional classification system that provides a dynamically up‐to‐date vocabulary. GO covers three ontologies: molecular function, cellular component, and biological process. First, this method mapped all DEGs to GO terms in the database (http://www.geneontology.org/), calculating the numbers of DEGs for every term. Then, a hypergeometric test was used to identify significantly enriched GO terms in the input list of DEGs. This analysis is able to recognize the main biological functions and processes that involve various DEGs. Pathway analysis is a functional analysis that maps genes to the Kyoto Encyclopedia of Genes and Genomes (KEGG) pathways. This analysis identifies metabolic or signal transduction pathways that are significantly enriched by DEGs.

### Quantitative RT‐PCR verification for target mRNAs

After filtering the results of the next‐generation sequencing, several target RNAs were selected for quantitative real‐time reverse transcription PCR (qRT‐PCR) validation. Eight samples of primary CRC with liver metastases and another eight without liver metastases were collected from the Department of Colorectal Surgery, the First Affiliated Hospital of Nanjing Medical University. For each sample, total RNA was isolated using the TRIzol (Invitrogen, Carlsbad, CA) and then reverse transcribed using the Prime Script RT reagent kit. QRT‐PCR was performed with SYBR green mix reagents on an ABI 7500 (Applied Biosystems, Carlsbad, CA), according to the manufacturer's instructions, to detect the expression levels of the target mRNAs.

### Immunohistochemistry analysis

Primary samples collected from patients with and without liver metastases were cut into 4‐*μ*m‐thick serial sections. The sections were submerged in antigenic retrieval buffer and microwaved for antigen fixation, after which they were deparaffinized with xylene. Slides were treated with hydrogen peroxide to quench endogenous nonspecific binding activity. The slides were then probed with the following primary antibodies: rabbit anti‐human SPP1, rabbit anti‐human MMP3, rabbit anti‐human OSM, rabbit anti‐human SLC13A2, and mouse anti‐human CXCL8 (all Proteintech, Inc. Wuhan, China); rabbit anti‐human HOXD10 (Bioss, Inc. Beijing, China), and rabbit anti‐human CXCL6 (Abcam, Cambridge, UK), respectively. Next, the slides were incubated with horseradish peroxidase (HRP)‐polymer‐conjugated secondary anti‐rabbit or anti‐mouse antibody at 37°C for 1 h. Then, the immunoreactivity was detected with the diaminobenzidine (DAB) substrate to determine protein expression. A negative control was performed by replacing the primary antibodies with phosphate‐buffered saline (PBS). The stained tissue slices were reviewed and scored by two pathologists separately.

## Results

### Overview

Six primary CRC samples were included in the NGS‐based profiling, generating approximately 12,807,481 raw sequencing reads, of which 12,782,782 clean reads remained after filtering low‐quality samples, making the average clean reads ratio 99.81% (Table 2). After Quality control (QC), clean reads are mapped to reference using BWA 18 and Bowtie2 19 tools. The average mapping ratio with reference genes was 74.48%. Table 3 lists separate mapping rates for each sample.

**Table 2:** Summary of sequencing data

Sample	Sequencing strategy	Raw data size (bp)	Raw reads number	Clean data size (bp)	Clean reads number	Clean data rate (%)
T2	SE49	639,113,762	13,043,138	638,528,996	13,031,204	99.9
T8	SE49	611,143,876	12,472,324	610,085,427	12,450,723	99.82
T9	SE49	613,761,652	12,525,748	612,491,866	12,499,834	99.79
T4	SE49	639,188,046	13,044,654	636,882,547	12,997,603	99.63
T6	SE49	639,151,394	13,043,906	638,229,557	13,025,093	99.85
T7	SE49	623,040,733	12,715,117	621,919,564	12,692,236	99.82

Strategy to sequence samples with paired‐end (PE); the following number reflects the read length.

**Table 3:** Alignment statistics of reads aligned to the reference gene

Sample	Total reads	Total bases	Total mapped reads (%)	Perfect match (%)	Mismatch (%)	Unique match (%)	Multi‐position match (%)	Total unmapped reads (%)
T2	13,031,204	638,528,996	72.43	62.27	10.15	63.82	8.6	27.57
T8	12,450,723	61,008,5427	75.17	61.7	13.47	66.75	8.42	24.83
T9	12,499,834	612,491,866	73.37	60.03	13.34	64.78	8.59	26.63
T4	12,997,603	636,882,547	77.59	63.28	14.31	68.74	8.86	22.41
T6	13,025,093	638,229,557	73.69	63.53	10.15	65.09	8.6	26.31
T7	12,692,236	621,919,564	74.62	61.19	13.44	65.98	8.65	25.38

**Figure 1:**
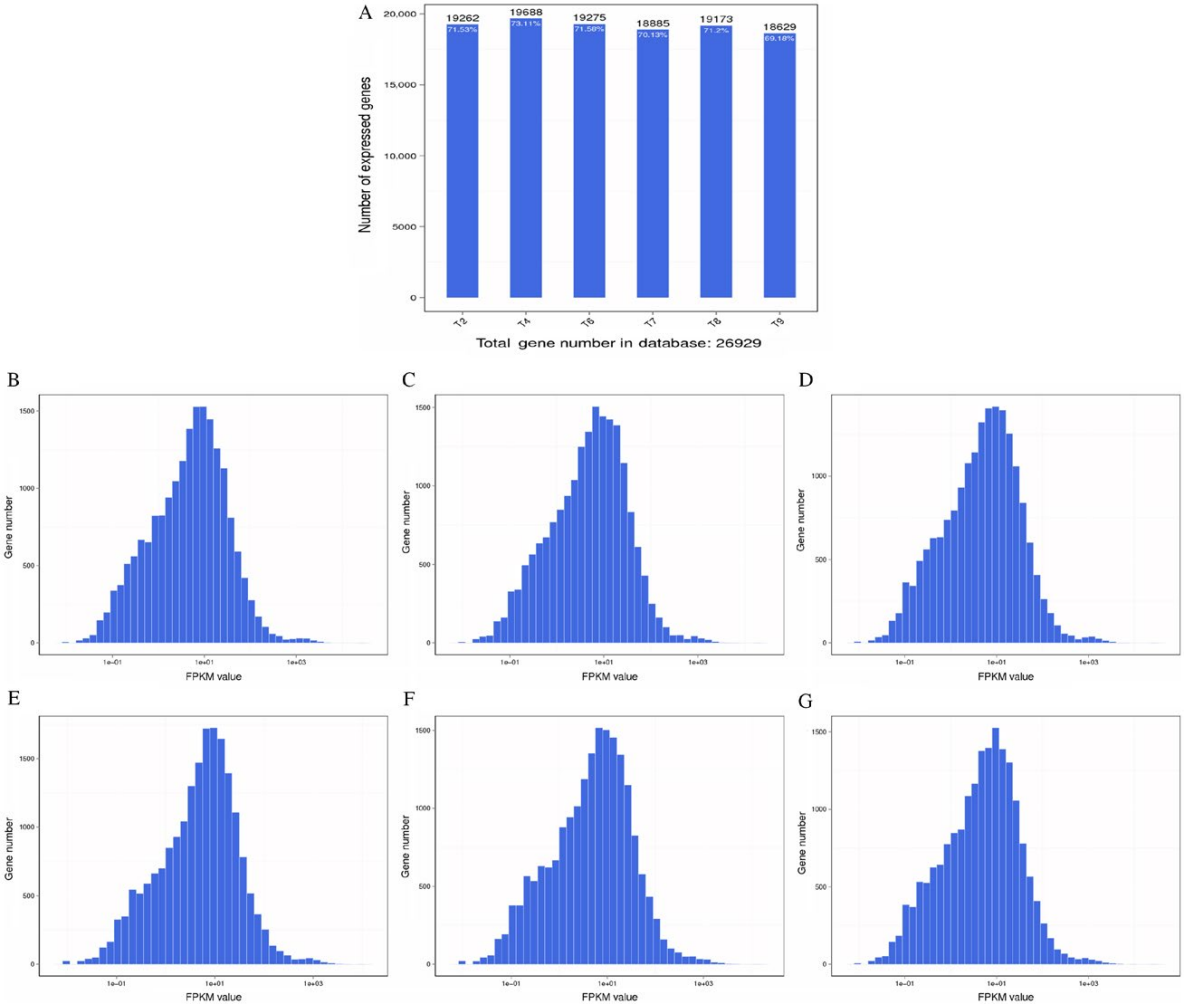
Analyses of identified genes (A) Number of identified genes. The *x*‐axis is the sample name. The *y*‐axis is number of identified expressed genes. The proportion at the top of each bar equals the number of expressed genes divided by the total gene number reported in database. (B–G) Histograms showing the distribution of genes at the expression level of T2, T8, T9, T4, T6, and T7. The *x*‐axis is fragments per kb per million fragments (FPKM) value. The *y*‐axis is gene number of at the corresponding FPKM.

Gene expression levels were quantified by a software package called RSEM 20. We counted the number of identified expressed genes and calculated their proportion to total number of genes (26,929 in total) in the database for each sample, as shown in Figure 1A. Also, histogram distributions were performed for each sample based on gene expressions. The average FPKM values were 24.76, 22.26, 24.69, 21.53, 22.84, and 24.42 for the T2, T8, T9, T4, T6, and T7 samples, respectively (Fig. 1B–G).

**Figure 2:**
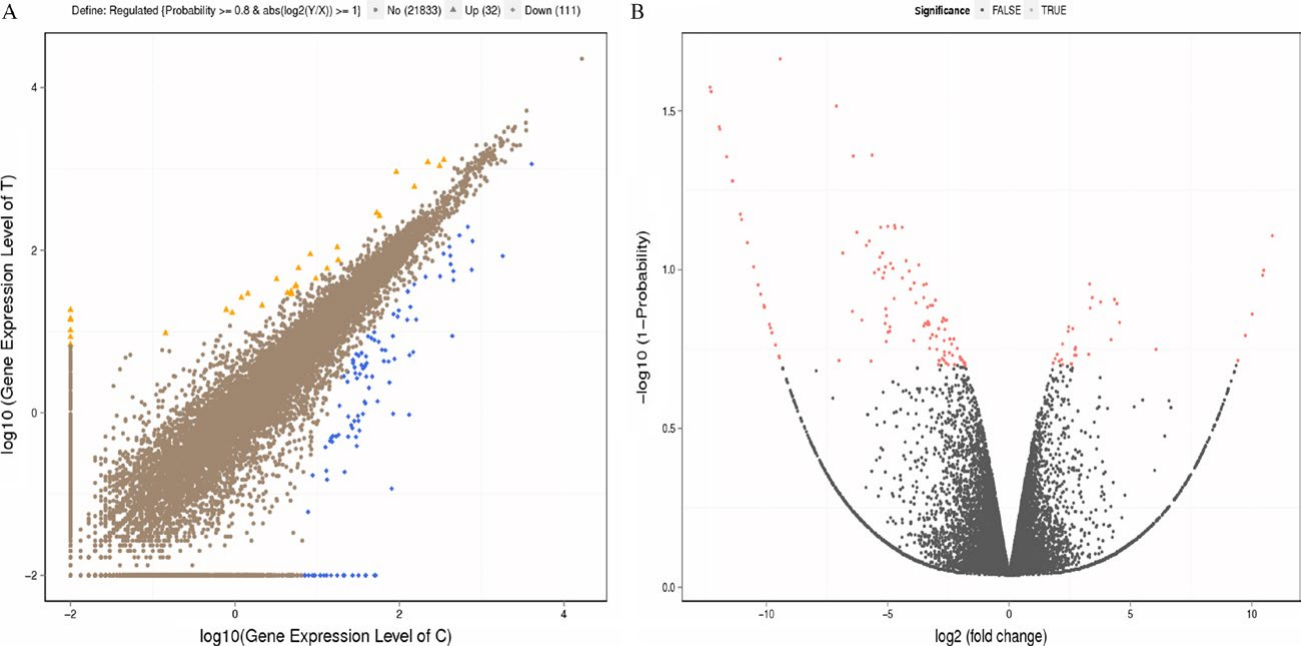
Scatter plots and Volcano graphs of all expressed genes. (A) Scatter plots of all expressed genes in each pairwise comparison. The *x* and *y*‐axes represent the log 2 value of gene expression for treatment (T) and control (C) groups. Blue means a downregulated gene, orange means an upregulated gene, and brown means a nonregulated gene. The screening threshold is shown on the top legend. (B) Volcano graph of all expressed genes in each pairwise comparison. The *x*‐axis shows the log 2 (fold change) and the *y*‐axis shows the –log 10 (1−*P*), representing the threshold values in log transformation. Each dot is a differentially expressed gene (DEG). Red dots mean significant DEGs and black dots are nonsignificant DEGs.

The whole‐transcriptome analysis revealed the differences between Treatment (T) Group and Control (C) Group. The 2690 dysregulated RNAs (log 2 ratio ≥2.0‐fold change) comprised 996 upregulated and 1694 downregulated DEGs. In addition, probability analysis reveals the possibility of how significantly these genes are differentially expressed between groups. The resulting range narrowed a lot at a probability ≥0.8, identifying of 32 upregulated and 111 downregulated DEGs (log 2 ≥ 2.0‐fold change, *P* < 0.05, *P* ≥ 0.8) (Fig. 2A and B). We then removed genes with extremely low FPKM (FPKM < 0.5) values in both the T and C groups on that basis 17, 21. Therefore, 22 upregulated and 73 downregulated DEGs were included after filtering (Table S1). Among the upregulated genes, *HOXD10* was the most upregulated DEG with a 4.56‐fold change, whereas the most downregulated DEG was *SPP1*, with a −7.12 fold‐change.

### Gene ontology and pathway analyses

**Figure 3:**
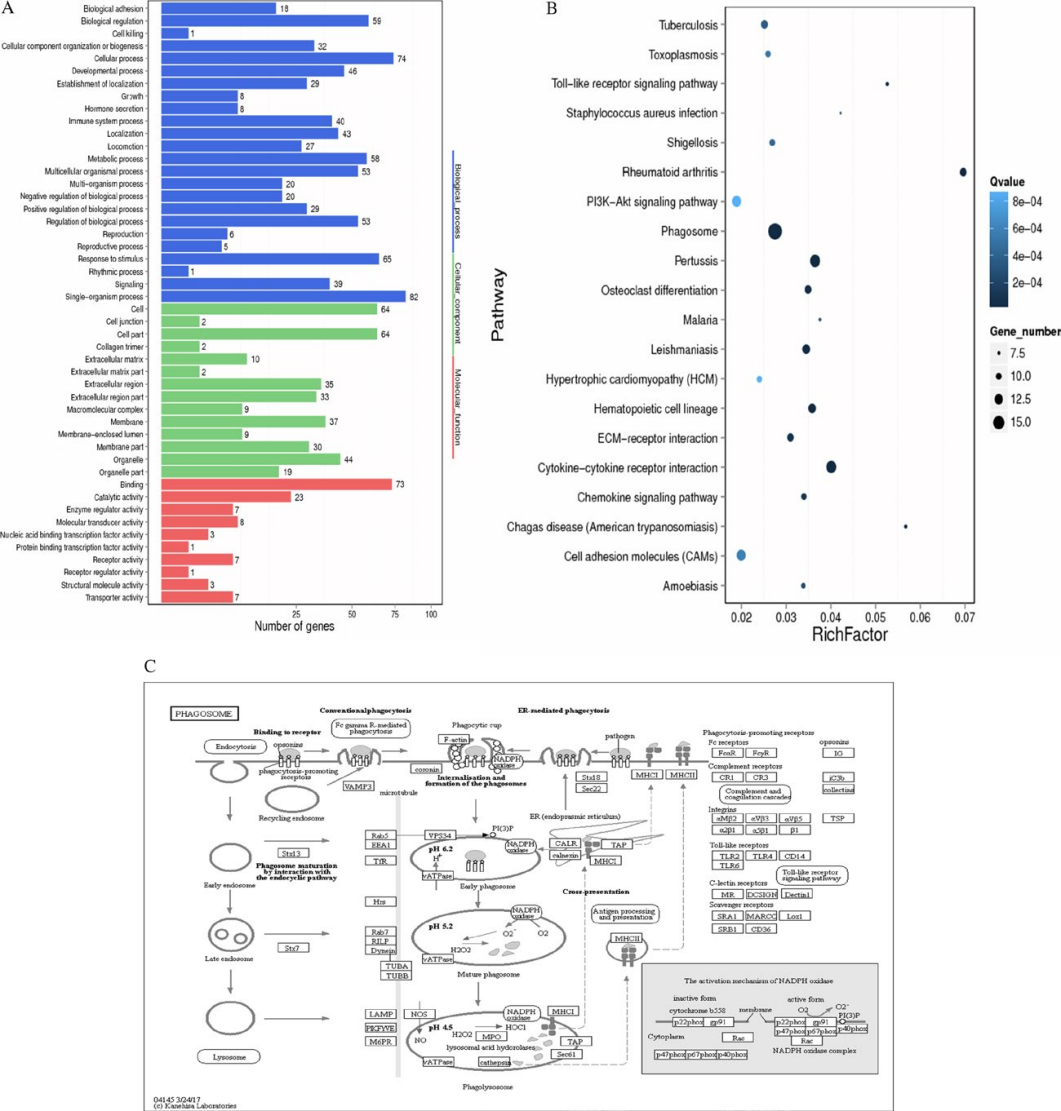
Functional analysis of differentially expressed genes (DEGs). (A) Gene ontology (GO) functional classification on DEGs between groups. The *x*‐axis represents the number of DEGs (the number is presented by its square root value). The *y*‐axis represents the GO terms. All GO terms are grouped into three ontologies: blue represents biological process, brown is cellular component, and orange is molecular function. (B) The Rich Factor is the ratio of DEGs numbers annotated in this pathway term to all gene numbers annotated in this pathway. A higher Rich Factor means greater intensiveness. The *Q*‐value is the corrected *P*‐value ranging from 0 to 1, and a smaller *Q*‐value means greater intensiveness. (C) The phagosome pathway is annotated with most DEGs among all the referred pathways.

In the GO analysis, the DEGs were annotated to different terms and grouped into three categories: biological process, cellular component, and molecular function. Between T and C groups, the three terms most associated with biological process were single‐organism process (82), cellular process (74), and response to stimulus (65). In the cellular component category, cell (64), cell part (64), and organelle (44) were the three most substantial components. Among molecular functions, binding (73) ranked first, followed by catalytic activity (23) and molecular transducer activity (8) (Fig. 3A).

The pathway enrichment analysis reveals the way in which DEGs would interact with other factors and how they participate in the various biological functions. Between the two groups, the top 10 pathways annotated with higher numbers of DEGs among all the dysregulated genes were: Phagosome (17), Cytokine‐cytokine receptor interaction (14), Pertussis (14), Cell adhesion molecules (CAMs) (13), PI3K‐Akt signaling pathway (13), Hematopoietic cell lineage (12), Leishmaniasis (12), Proteoglycans in cancer (12), Rheumatoid arthritis (11), and ECM‐receptor interaction (11). All pathways displayed in Figure 3B reached statistically significance (*P* < 0.05). The pathway involving the most DEG annotations is displayed in Figure 3C.

### Hierarchical clustering analysis of DEGs

**Figure 4:**
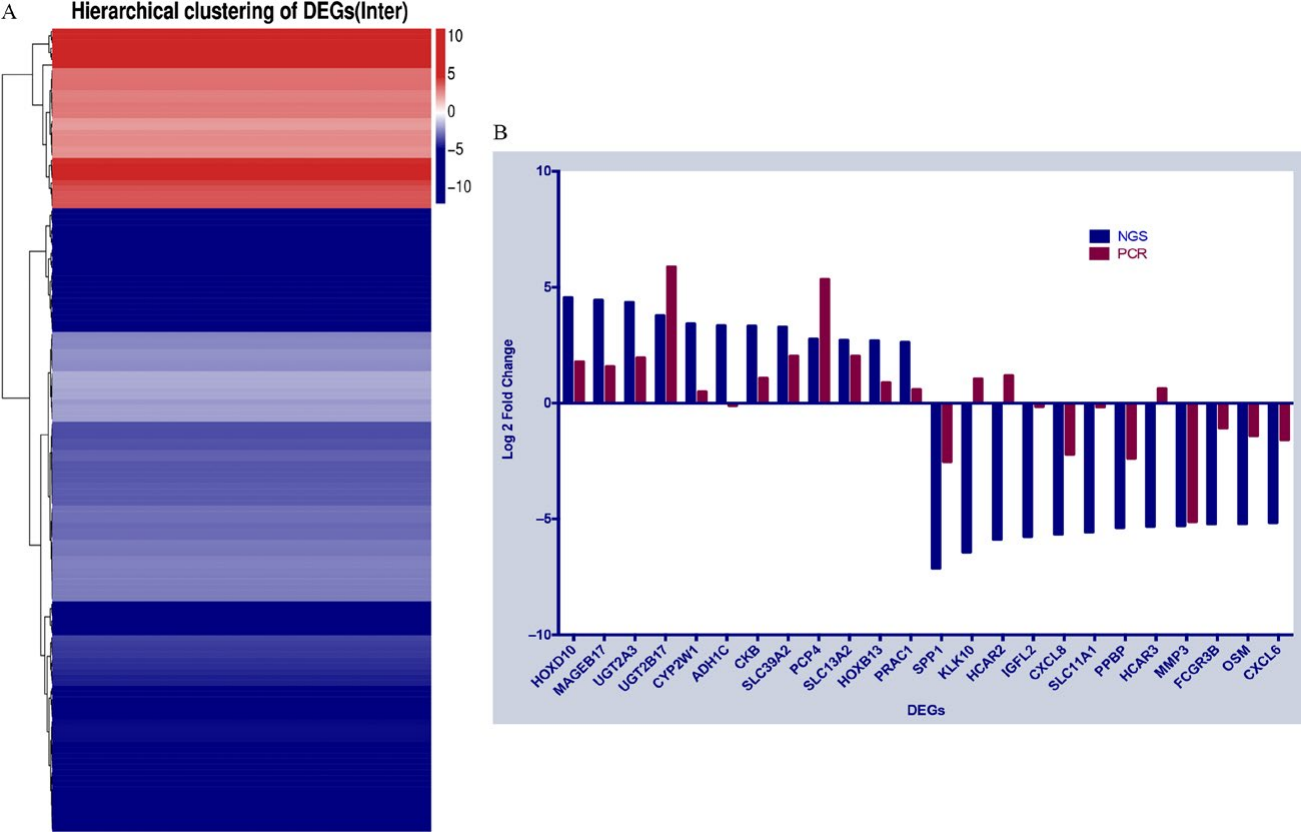
Analysis of differentially expressed genes (DEGs). (A) Only genes that were differentially expressed in all pairwise comparisons of the cluster plan were used to build this heatmap. The gradient color barcode at the right top indicates the log 2 fold change value. Each row represents a DEG and each column represents a pairwise condition. DEGs with similar fold changes value are clustered both at the row and column level. (B) Comparison of the expressions obtained using two platforms showed good correspondence for most pairs. The blue columns represent the log 2 ratio fold change in each DEG with the application of next‐generation sequencing (NGS) and red columns represent log 2 fold changes using quantitative real‐time reverse transcription PCR (qRT‐PCR).

Genes with similar expression patterns usually have functional correlations. Therefore, clustering analysis of DEGs was performed using Cluster 22, according to the provided cluster plans for DEGs. We also provided a complete intersection and union DEGs heatmap (Fig. 4A) for each cluster plan.

### Target DEGs selection and validation

After removing genes with extremely low FPKM values (FPKM < 0.5), we selected the top 13 upregulated and top 12 downregulated genes by fold change to verify their differential expression using qRT‐PCR. These target DEGs are listed in Table 4. QRT‐PCR validated that eight of the 25 selected DEGs demonstrated significant changes between groups. The three most significantly upregulated DEGs were *HOXD10*,* UGT2A3*, and *SLC13A2*, whereas the five most significantly downregulated DEGs were *SPP1*,* CXCL8*,* MMP3*,* OSM*, and *CXCL6*, respectively. The most up‐ and downregulated DEGs in the list reached statistical significance. A comparison was also performed between outcomes of NGS and qRT‐PCR, as shown in the Figure 4B and Table 4. Good correlations of expressions were observed in most pairs between the two platforms, demonstrating the credibility of these results.

**Table 4:** Validation outcomes of target genes via next‐generation sequencing (NGS) and quantitative real‐time reverse transcription PCR (qRT‐PCR) separately

Symbol	Regulation (T/C)	Log 2 ratio (T/C) (NGS)	Probability (T/C)	*P*‐value (PCR)	Significance (PCR)	Log 2 ratio (T/C) (PCR)
HOXD10	UP	4.56	0.85	0.0399	*	1.7818003
MAGEB17	UP	4.44	0.87	0.2719	NS	1.5873628
UGT2A3	UP	4.35	0.88	0.0231	*	1.9476197
HOXD11	UP	4.20	0.83	*N*	NS	*N*
UGT2B17	UP	3.77	0.87	0.2305	NS	5.8695526
CYP2W1	UP	3.43	0.88	0.6263	NS	0.49270165
ADH1C	UP	3.34	0.87	0.9272	NS	−0.10134707
CKB	UP	3.32	0.89	0.2883	NS	1.066491
SLC39A2	UP	3.28	0.82	0.4047	NS	2.0332544
PCP4	UP	2.77	0.81	0.1535	NS	5.333551
SLC13A2	UP	2.72	0.82	0.0088	**	2.0332544
HOXB13	UP	2.69	0.81	0.1130	NS	0.88917387
PRAC1	UP	2.62	0.85	0.6127	NS	0.58156765
SPP1	DOWN	−7.12	0.97	0.0055	**	−2.5303776
KLK10	DOWN	−6.43	0.96	0.3147	NS	1.0371485
HCAR2	DOWN	−5.88	0.92	0.0572	NS	1.1890949
IGFL2	DOWN	−5.76	0.92	0.0545	NS	−0.13972865
CXCL8	DOWN	−5.65	0.96	0.0073	**	−2.2027724
SLC11A1	DOWN	−5.56	0.90	0.7785	NS	−0.154957
PPBP	DOWN	−5.37	0.90	0.3707	NS	−2.3749266
HCAR3	DOWN	−5.32	0.91	0.3892	NS	0.61707544
MMP3	DOWN	−5.29	0.93	0.0301	*	−5.1104937
FCGR3B	DOWN	−5.21	0.91	0.1368	NS	−1.0682427
OSM	DOWN	−5.20	0.89	0.0437	*	−1.4058526
CXCL6	DOWN	−5.15	0.90	0.0392	*	−1.5858094

NS means not significant and statistical significance (*) is defined as *P* < 0.05.

**P<0.01

### Immunohistochemistry validation of the expressions of target genes

**Figure 5:**
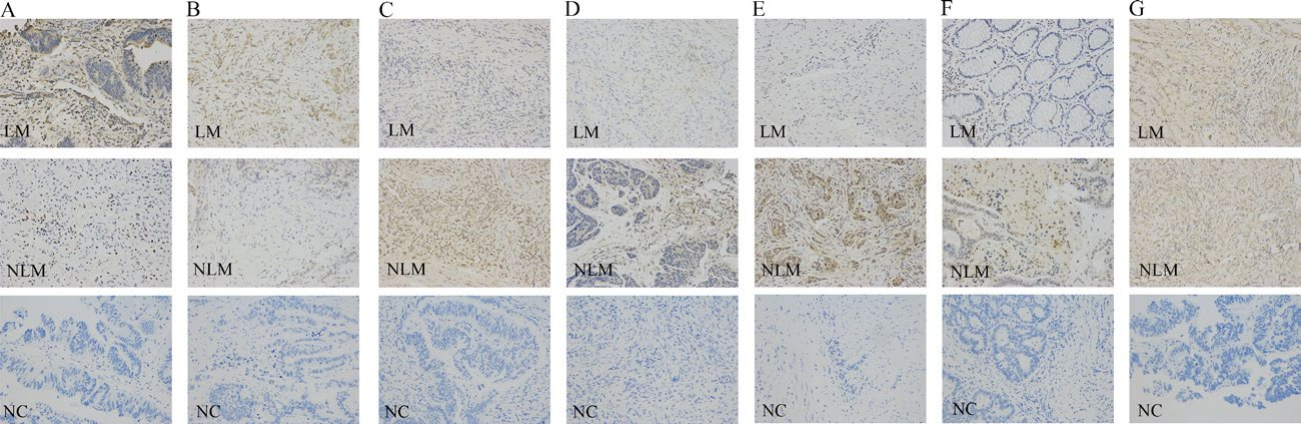
IHC validation for HOXD10 (A), SLC13A2 (B), OSM (C), MMP3 (D), CXCL6 (E), CXCL8 (F), and SPP1 (G). For each gene: LM represents primary CRC samples with liver metastases; NLM represents primary CRC samples without liver metastases; NC represents negative control; original magnification: ×200.

Immunohistochemistry (IHC) validations were performed on primary CRC samples with and without liver metastases. Among the upregulated DEGs, IHC for HOXD10 and SLC13A2 showed higher levels in primary samples with liver metastases (LM) than in those without liver metastases (NLM) (Fig. 5A and B). Notably, since anti‐human UGT2A3 could not be applied in IHC according to the Abcam official instructions, we did not perform IHC to detect UGT2A3 protein levels. For the downregulated DEGs, OSM, MMP3, CXCL6, and CXCL8 all displayed expression patterns similar to the qRT‐PCR validations; that is, the protein levels were lower in the primary CRC tissues with liver metastases than in those without liver metastases (Fig. 5C–F). The IHC staining on tissues with liver metastases with cytokine CXCL6 and CXCL8 antibodies were extremely weak, demonstrating a strong distinction compared to those in tissues without liver metastases. However, IHC analysis between tissues using SPP1 antibodies demonstrated no significant difference (Fig. 5G). Collectively, the outcomes of the IHC analyses were mostly consistent with the qRT‐PCR results, except for SPP1.

## Discussion

Liver is the most frequent organ to form distant metastases in CRC, and liver metastases are the leading cause of death for patients with CRC. Therefore, it is important to explore the underlying mechanism and detect novel biomarkers of liver metastases. Several microarray studies revealed that liver metastases sites demonstrated a high correspondence 17 with their primary tumor in terms of gene expression; therefore, we adopted the strategy whereby primary tumors from patients with and without liver metastases were selected to detect gene expressions via NGS, which avoided the potential bias caused by different organs. Moreover, we selected only left colon tumors as samples to rule out the bias from different locations of CRC, because the new NCCN guidelines of colon cancer have already mentioned that anti‐EGFR therapy has relatively lower benefits on the right‐sided colon compared with the left‐sided colon. Our results revealed that the potential DEGs might contribute to or inhibit the process of liver metastases.

*HOXD10* was the most upregulated DEG in this study. HOX genes are a family of regulators that play important roles in organ and cell development during embryogenesis. Interestingly, studies have revealed that HOXD10 affects the outcomes of different tumors. According to Sharpe and colleagues 23, HOXD10 and HOXD11 are expressed at high levels in head and neck squamous cell carcinoma, which corresponded to our results. Knockdown of *HOXD10* led to decreased migration and proliferation in head and neck squamous cell carcinoma cells in their study, which suggested that HOXD10 acts as an oncogene that promote metastases. Interestingly, studies concerning breast cancer and gastric cancer showed opposite effects. According to Wang and colleagues 24, HOXD10 is downregulated in gastric cancer tissues and cell lines compared with normal tissues, suggesting that high expression of HOXD10 impaired cell migration and invasion, which may function as tumor suppressor. In breast cancer, significant differences were found between cancerous and normal tissues, and low expression of HOXD10 was associated with high‐grade breast cancer, displaying a similar tumor suppressor function to that in gastric cancer 19. In our study, *HOXD10* was expressed at a significantly higher level in primary CRC with liver metastases than in those without liver metastases, indicating that our results favor the identification of *HOXD10* as an oncogene in colorectal cancer. This might need further validation.

The UDP‐glucuronosyltransferase (UGT) enzymes are responsible for the glucuronidation of exogenous and endogenous compounds, including drugs, environmental intoxicants, steroids, neurotransmitters, bile acids, and other hormones. UGT2A3 is one of the UGT family members. It is reported that UGT2A3 expressed at its highest levels in tissues that are mostly related to drug clearance, of which liver is the most expressed organ, followed by the gastrointestinal tract, and the kidneys. Also, UGT2A3 was found to glucuronidate bile acid specifically, which is a product of cholesterol metabolism. Normally, 90% of bile acids that pass through the biliary system would be reabsorbed and returned to the liver. Therefore, if the bile flow is somewhat impaired or limited by cholestatic liver disease, liver injuries would occur due to the accumulation of bile acids. In our study, primary CRC patients with liver metastases showed significantly higher levels of UGT2A3 than those without liver metastases. The liver dysfunction caused by liver metastases may influence the normal bile flow, therefore leading to cholestasis. The accumulation of bile acids requires higher UGT2A3 activity to catalyze the process of glucuronidation. This hypothesis highlights the significance of metabolism in carcinogenesis, as reported by Lindsey and colleagues 25. We hypothesized that UGT2A3 could be a potential biomarker of CRC with liver metastases 26.

Like UGT2A3, few reports have focused on SLC13A2. SLC13A2 encodes the Na^+^‐dicarboxylate cotransporter isoform 1 (NaDC1), which plays an important role in dealing with renal citrate. Mostly, NaDC1 is located on the apical membrane of epithelial cells of the renal proximal tubule and small intestine, where absorption of citric acids take place 27. In the kidney, the function of NaDC1 is involved with formation of kidney stones because of the existence of divalent citrate (citrate^2+)^. Although it seems irrelevant to CRC with liver metastases, our results showed that SLC13A2 is associated with liver metastases, although the underlying mechanism has yet to be determined.

Among the downregulated DEGs, OSM and SPP1 shared one the same pathway, the PI3K‐Akt pathway, indicating that this pathway is might be involved in tumorigenesis and metastases. SPP1, also known as OPN, activates the PI3K/Akt signaling pathway through an extracellular matrix (ECM)‐Receptor interaction, whereas OSM activates the pathway via a cytokine‐cytokine receptor interaction. Matrix metalloproteinase‐3 (MMP‐3) is a member of the MMP family that is associated with several kinds of malignant tumors 28. The CXCR‐1 binding chemokines IL‐8/CXCL8 and CXCL6 are often coinduced with CXCL6, according to several reports 29, 30, resulting in the same trend in regulation. Interestingly, it is reported that CXCL6 staining correlates with MMP9 expression in gastrointestinal malignancies. In contrast, another study reported that CXCL6 suppressed the activity of MMP2 in the first trimester, indicating various functions and roles of CXCL6 31. In addition, with regard to CXCL8, it is reported that a high serum level of CXCL8 is a protective factor to prevent metastases, and high CXCL8 levels are associated with better prognosis 32. Meanwhile, some studies 33, 34 show that CXCL8 promotes cancer progression and is linked to poor prognosis. Thus, it still remains controversial how CXCL8 would influence the metastases; however, our results showed that reductions in the expression of CXCL8 is associated with tumor progression and metastases, indicating that these cytokines could possibly play roles as tumor suppressors and biomarkers. Both CXCL6 and CXCL8 showed diverse expression between them, by qRT‐PCR and IHC analysis, and both methods demonstrated a great correspondence in all samples.

Advanced bioinformatics tool could help us to understand the roles of these DEGs. In terms of Gene Ontology, one of the top terms associated with biological process is single‐organism process, which agrees with a previous study by Chen and colleagues 35, whereas in the molecular function analysis, binding is another term shared between this study and the previous study 35. This consistency not only suggests that most DEGs contribute to these two common processes, but also enhanced the credibility of both studies. For the KEGG pathways, cytokine‐cytokine receptor interaction, CAMs, PI3K‐Akt signaling pathway, Leishmaniasis, and rheumatoid arthritis were also identified in the previous study 35. Among these, the PI3K‐Akt pathway is recognized as a carcinogenesis‐related pathway, which is also the pathway shared by two validated DEGs, OSM and SPP1, suggesting that the PI3K‐Akt pathway is very significant 36, 37. Cytokine‐cytokine receptor interaction is often associated with carcinogenesis and metastases of CRC 38, 39, 40, 41, which also corresponds to our observation of significantly differentially expressed cytokines CXCL6 and CXCL8. CAMs also ranked high among all these pathways because the process of cells migration to distant sites is associated with adhesion ability and the epithelial‐mesenchymal transition (EMT)^42^, ^43^, ^44^, which is also highly correlated with “binding” in GO molecular function. We believe that these pathways might play more important roles in the metastases as well as in carcinogenesis.

Obviously, one of the disadvantages of this study is that number of samples for NGS is relatively small. More qualified samples are required to perform large‐sized NGS‐based whole‐transcriptome profiling. Moreover, functional validation of these DEGs is also required for the connections between some DEGs and carcinogenesis and metastases. Lastly, since there is abundant information for numerous DEGs, more valuable results could be obtained from the primary statistics.

Collectively, the goal of this study was to discover DEGs that might be responsible for liver metastases or could be biomarkers of liver metastases, using NGS‐based whole‐transcriptome profiling on CRC tissues with and without metastases. Validations of the top 25 up and downregulated DEGs were performed via qRT‐PCR and IHC. The results showed that these DEGs were significantly differentially expressed and corresponded to the previous NGS profile. To the best of our knowledge, this is the first study to perform NGS‐based mRNA whole‐transcriptome on CRC tissues with and without liver metastases. These findings might provide novel targets for colorectal liver metastases and biomarker discovery, suggesting potential roles in therapeutic application.

## Conflict of Interest

The authors declare that they have no competing interests.

## Supporting information


**Table S1.** Potential target DEGs
